# Stillbirth rates and their association with swine leucocyte antigen class II haplotypes in Microminipigs

**DOI:** 10.5713/ab.20.0763

**Published:** 2021-06-23

**Authors:** Noriaki Imaeda, Asako Ando, Tatsuya Matsubara, Masaki Takasu, Naohito Nishii, Asuka Miyamoto, Shino Ohshima, Yoshie Kametani, Shingo Suzuki, Takashi Shiina, Tetsushi Ono, Jerzy K. Kulski, Hitoshi Kitagawa

**Affiliations:** 1 Department of Veterinary Medicine, Faculty of Applied Biological Sciences, Gifu University, Gifu 501-1193, Japan; 2 Department of Molecular Life Science, Division of Basic Medical Science and Molecular Medicine, Tokai University School of Medicine, Isehara 259-1193, Japan; 3 Department of Veterinary Medicine, Faculty of Veterinary Medicine, Okayama University of Science, Imabari 794-8555, Japan; 4 Faculty of Health and Medical Sciences, UWA Medical School, The University of Western Australia, Crawley, WA 6009, Australia

**Keywords:** Breeding, Haplotypes, Microminipig, Stillbirth, Swine Leukocyte Antigen

## Abstract

**Objective:**

Microminipig (MMP) is a miniature pig with an extra small body size for experimental use. In the present study, the occurrence of stillbirths and their genetic association with swine leukocyte antigen (SLA) class II haplotypes were evaluated in a population of MMPs.

**Methods:**

The occurrences of stillbirth and genetic association with SLA class II haplotypes using 483 stillborn and 2,246 live piglets, and their parents were compared among the three groups of newborn piglet litters; an all stillborn (AS) group consisting of only stillborn piglet litters, a partial stillborn (PS) group consisting of stillborn and live piglet litters, and an all alive (AA) group consisting of only live piglet litters.

**Results:**

The incidence of stillborn piglets was 483/2,729 (17.7%). Distributions of litter sizes, numbers of stillborn piglets in a litter, parities, and gestation periods were distinct among the three groups. The frequencies of low resolution haplotype (Lr)-0.7 or Lr-0.23 were higher in the AS group than in the PS or AA groups. In sires, the frequency of Lr-0.7 associated with the AS group was significantly higher in the AS group than with the AA group. In dams, the frequency of Lr-0.23 was significantly higher in the AS group than in the PS or AA groups, whereas the frequency of Lr-0.7 was not significantly different.

**Conclusion:**

The incidence of stillborn piglets in MMPs appears to be higher than those in other pig breeds. Several traits related with stillbirths such as the number of stillborn piglets and parities of the AS group were different from those of the PS and AA groups. Specific SLA class II haplotypes were associated significantly with a high incidence of stillbirths and could be used as genetic markers to adopt breeding strategies to lower the rate of stillbirth in MMPs.

## INTRODUCTION

Stillbirth is defined as the birth of a dead fetus. Many factors such as maternal, piglet, environmental, and genetic factors have been associated with stillbirths [1]. According to the review by Vanderhaeghe et al [1], maternal factors are body condition, litter size, parity, gestation length and farrowing duration; piglet factors are birth interval, birth order, and birth weight; and environmental factors are nutrition, stress, insufficient ventilation and so on. In addition, there are differences in the incidence of stillbirth among the lines or breeds of pigs that may involve various complicated genetic factors [2,3]. Recently, genome wide association analyses were applied to studies of stillbirth events, and several candidate genes associated with increased stillbirth were mapped on a few chromosomal regions in maternal and terminal Landrace, Duroc, and Yorkshire lines [4]. However, the specific genes responsible for stillbirth have not been identified so far.

The major histocompatibility complex (MHC) molecules that present non-self and self-antigens to T cells and have crucial roles in inducing antigen-specific immune responses and preventing infectious diseases [5–7] might also have a direct or indirect role in stillbirth events. The MHC molecules influence various biological traits, such as immune recognition, autoimmunity, mating preferences, and pregnancy outcomes [8]. Furthermore, associations between specific swine leukocyte antigen (SLA) alleles or haplotypes and productive or reproductive traits such as ovulation rates, litter sizes, and gestation periods have been reported in several breeds of pigs [9–12].

Microminipigs (MMPs) were developed as miniature pigs with an extra-small body size for experimental use [13]. The population of MMPs are distinguished by eight different kinds of SLA class II haplotypes [14] that could be used as genetic markers for analyses of associations with specific phenotypes for various productive or reproductive traits and outcomes. In this regard, we previously reported associations of the SLA haplotypes with body weights [15] and some reproductive traits, such as gestation periods, fertility indices, litter sizes, and numbers of abnormal newborn piglets in MMPs [16]. Moreover, stillborn piglets in the MMP population are a serious recurring problem and the reduction of the risk factors is important for a more effective farrowing management of MMP and other commercial pigs. In the present study, to reduce stillbirths and the associated risk factors in the MMP population, we investigated relationships between stillbirth and factors such as litter sizes, parities, and gestation periods, as well as their genetic association with eight SLA class II haplotypes.

## MATERIALS AND METHODS

### Animals and groups examined

MMPs were bred as a herd at Fuji Micra Inc. (Fujinomiya, Japan). A total of 484 deliveries, and 2,729 newborn piglets of MMPs consisting of 483 stillborn and 2,246 live piglets were used for analyses of stillbirths. The examination period was from October 2012 to February 2017. All stillborn piglets were dead piglets with normal outward appearances at birth. Each litter of newborn piglets was classified into one of three birth (delivery) groups; the all stillborn (AS) group consisting of only stillborn piglets in a litter, the partial stillborn (PS) group consisting of both stillborn and born live piglets in a litter, and the all alive (AA) group consisting of only born live piglets in a litter. In the AS, PS, and AA groups, respectively, the number of deliveries were 31, 177, and 276, and numbers of newborn piglets were 120, 1,130 (consisting of 363 stillborn and 767 alive piglets), and 1,479. To clarify related factors with stillbirth, litter sizes, distributions of litter sizes and parities of dams, and gestation periods were compared among the three birth groups. The litter sizes were measured at birth as the total number of living and stillbirth newborn piglets in 484 deliveries. The gestation periods of continuous deliveries below 99 days after copulation were excluded from the data. SLA class II haplotype frequencies were compared among the AS, PS, and AA groups, and between the stillborn and live piglets in both the AS and PS groups. This study was approved by the Animal Care and Use Committee of Gifu University (No. 17042, May 26, 2017). The care and use of the laboratory animals were conducted in compliance with the guidelines of Good Laboratory Practice of Gifu University and Fuji Micra Inc.

### Swine leukocyte antigen class II haplotype typing and animals used

SLA class II-*DRB1* and *DQB1* alleles were assigned by low-resolution SLA genotyping using a polymerase chain reaction-sequence specific primers method as described previously [14]. Eight types of low-resolution SLA class II haplotypes, Lr-0.7, Lr-0.11, Lr-0.13, Lr-0.16, Lr-0.17, Lr-0.18, Lr-0.23, and Lr-0.37 were determined by an analysis of the inheritance and segregation of eight and four alleles of the *DRB1* and *DQB1* genes, respectively, in descendants of the MMP population.

The number of stillborn piglets analyzed were 110 in the AS group. In the PS group, numbers of stillborn and living piglets were 245 and 714, respectively. The number of newborn piglets was 1,358 in the AA group. In total, the number of piglets was 2,427 and the number of haplotypes analyzed was 4,854. SLA class II haplotype frequencies were also compared between the stillborn and live piglets: the number of stillborn piglets was 355 (710 haplotypes), 110 piglets in the AS group and 245 piglets in the PS group; whereas the number of live piglets was 2,072 (4,144 haplotypes), 714 piglets in the PS group and 1,358 piglets in the AA group. The parents of these piglets, 48 sires and 114 dams, also were assigned SLA class II haplotypes. Cumulative total numbers of sires in the AS, PS, and AA groups were 28, 175, and 268, respectively. The number of dams in the three groups were 30, 177, and 273, respectively.

### Statistical analysis

The data are expressed as median and ranges (minimum to maximum) or percentages of total deliveries. Statistical comparisons were carried out by multiple group comparison with Kruskal-Wallis and Scheffe’s F tests (BellCurve in Excel, Social Survey Research Information Co., Ltd. Tokyo, Japan). Pairwise comparisons were adjusted for multiple tests with a Bonferroni correction. Data are expressed as (Q1–1.5 IQR)-Q1-Med-Q3-(Q3+1.5IQR) in the box-and-whisker plot. The Q1, Med, Q3, and IQR indicate the first quartile, median, third quartile, and interquartile range, respectively. Distributions of litter sizes and parities were evaluated by the Chi-square for independence test, using an m×n contingency table.

## RESULTS

### Stillbirth occurrence in Microminipigs

Percentage of total deliveries in the AS, PS, and AA groups were 6.4% (31 deliveries of all 484 ones used in this study), 36.6% (177 deliveries), and 57.0% (276 deliveries), respectively. Percentages of newborn piglets in each group were 4.4% (120 piglets of all newborn ones used in this study), 41.4% (1,130 piglets), and 54.2% (1,479 piglets), respectively, and numbers of stillborn piglets in the AS and PS groups were 120 and 363, respectively. Incidences of stillborn piglets were 32.1% in the PS group, and 17.7% for total of newborn piglets in this population of MMPs.

**Figure 1:**
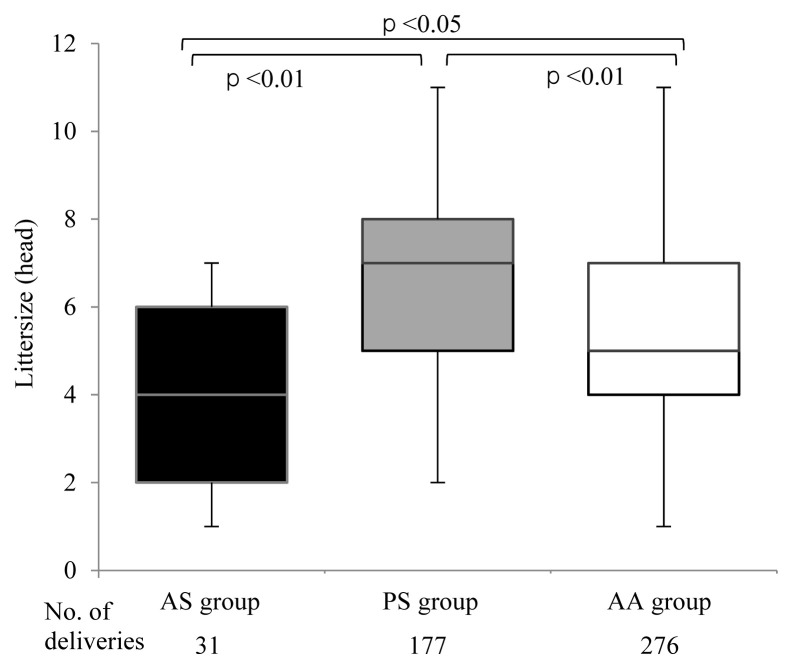
Comparison of litter sizes among three groups, AS, PS, and AA in MMPs. AS, all stillborn; PS, partial stillborn; AA, all alive; MMP, Microminipig. The data are expressed as (Q1–1.5 IQR)-Q1-Med-Q3-(Q3+1.5IQR) in the box-and-whisker plot.

In Figure 1, the median and range (minimum to maximum) of litter sizes in the AS, PS, and AA groups were 4 (1–7), 7 (2–11), and 5 (1–11), respectively. The median of litter sizes in the PS group was significantly larger than that in the AS (p<0.01) or AA groups (p<0.01). The number of stillborn piglets in the PS group tended to increase proportionally as the litter sizes increased (data not shown).

**Figure 2:**
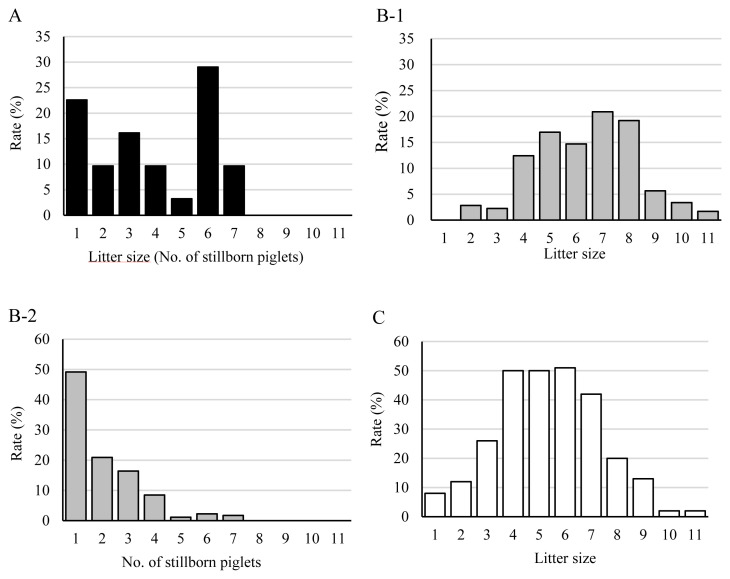
Distributions of litter sizes and numbers of stillborn piglets in the AS, PS, and AA groups. (A) Distribution of litter sizes in the AS group. Rate (%) on Y axis indicates the percentage of deliveries for each litter size from a total of 31 deliveries of only stillborn piglets. (B-1) Distribution of litter sizes in the PS group. Rate (%) on Y axis indicates the percentage of deliveries for each litter size from a total of 177 deliveries of both stillborn and live piglets. (B-2) Distribution of number of stillborn piglets in the PS group. Rate (%) on Y axis indicates the percentage of deliveries for each number of stillborn piglets in a litter from a total of 177 deliveries consisting of live and stillborn piglets. (C) Distribution of litter sizes in the AA group. Rate (%) on Y axis indicates the percentage of deliveries for each litter size from a total of 276 deliveries of only live piglets. AS, all stillborn; PS, partial stillborn; AA, all alive.

Figure 2 shows distribution patterns of the litter sizes in groups AS, PS, and AA and the relative number of stillborn piglets in groups AS and PS. The data are shown as the percentage of deliveries (y-axis) in each litter size with stillborn piglets in relation to the total number of deliveries. In the AS group, the highest percentage of deliveries was 29% in the group with six piglets per litter, and the plot of percentage of deliveries versus litter size was not a normal distribution (Figure 2A). In contrast, the rates of litter sizes in the PS group showed a relatively normal distribution pattern (Figure 2B-1). On the other hand, Figure 2B-2 shows a negative relationship between the rate (%) and the number of stillborn piglets. The percentage of deliveries with stillborn piglets showed a progressive reduction as the number of piglets in each litter increased (Figure 2B-2). Litter sizes also showed a normal distribution pattern in the AA group, as well as that in the PS group (Figure 2C). Thus, the distribution pattern for the litter sizes in the PS and AA groups per percentage of deliveries were similar, whereas the distribution pattern of litter sizes in the AS group that only consisted of stillborn piglets was markedly different from those in the PS and AA groups.

Table 1 shows the distribution of the number and percent age of deliveries in each parity for the three groups, AS, PS, and AA. In comparing the rates of parities in the AS group, the largest number of deliveries as a percentage were found in the 1st and 2nd parities and then the number of deliveries after the third parity tended to decrease and vary irregularly. The distribution pattern of parities in the PS and AA groups showed relatively high rates in low parities and decreased gradually as the parities increased.

**Figure 3:**
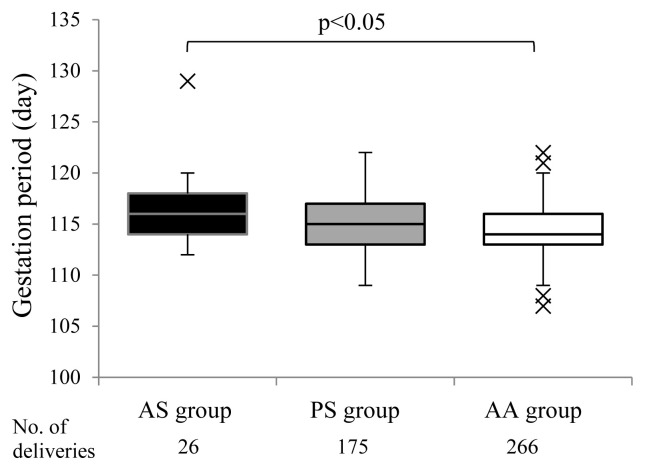
Comparison of gestation periods among three groups in MMPs. The data are expressed as (Q1-1.5 IQR)-Q1-Med-Q3-(Q3+1.5IQR) in the box-and-whisker plot. MMP, Microminipig.

**Figure 4:**
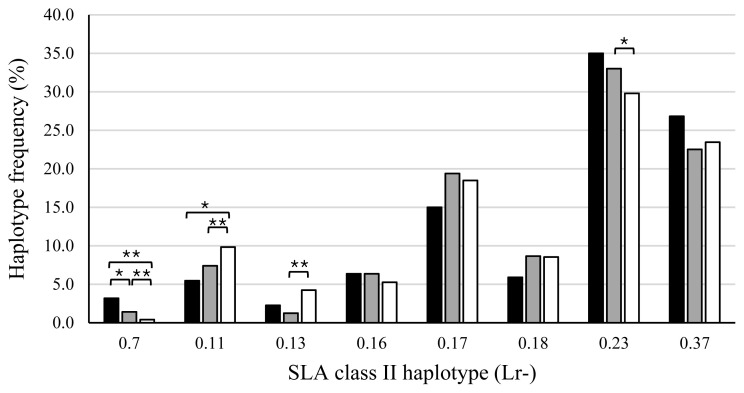
Comparison of swine leukocyte antigen (SLA) class II haplotype frequencies among piglets of the AS, PS, and AA groups. X-axis shows the haplotypes of homozygous or heterozygous piglets. AS, all stillborn; PS, partial stillborn; AA, all alive. ■, AS group (the number of haplotypes is 220); ■, PS group (the number of haplotypes is 1,918); □, AA group (the number of haplotypes is 2,716). * p<0.05, and ** p<0.01.

Figure 3 show a comparison of gestation periods among three groups AS, PS, and AA. The median of gestation periods in the AS group (116 [112–129] days) was significantly longer than that in the AA group (114 [107–122] days, p<0.05). The median of gestation periods in the PS group (115 [109–122] days) was intermediate between those in the AS and AA groups (Figure 4).

### Swine leukocyte antigen class II haplotype frequencies of piglets in the AS, PS, and AA groups

**Figure 5:**
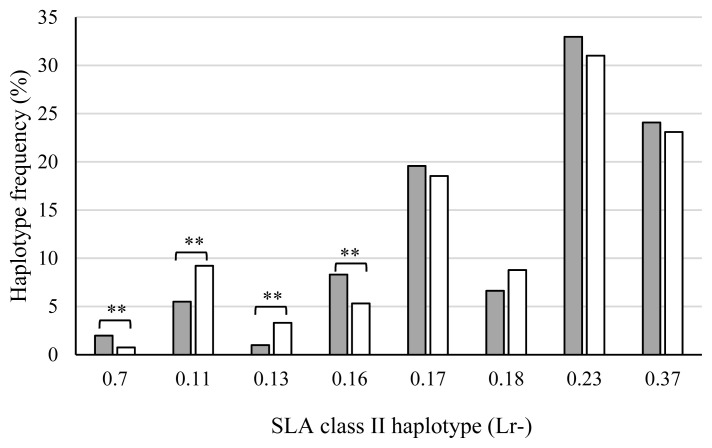
Comparison of swine leukocyte antigen (SLA) class II haplotype frequencies between the stillborn and live piglets. X-axis shows the haplotypes of homozygous or heterozygous piglets. ■, Stillborn piglet group (the number of haplotypes is 710); □, live piglet groups (the number of haplotypes is 4,144). ** p<0.01.

Figure 4 shows the SLA class II haplotype frequencies of piglets in the AS, PS, and AA groups. The haplotype frequency of Lr-0.7 in the AS group was significantly higher than that in the PS (p<0.05) or AA (p<0.01) groups, and the frequency in the PS group was also significantly higher than that in the AA group (p<0.01). The frequencies of Lr-0.11 in the AS (p<0.05) and PS (p<0.01) groups were lower than that in the AA group. Furthermore, the haplotype frequency of Lr-0.13 in the PS group was lower than that in the AA group (p<0.01). In contrast, the frequency of Lr-0.23 in the PS group was higher than that in the AA group (p<0.05). SLA class II haplotype frequencies were compared between the stillborn and live piglets of the AS and PS groups (Figure 5). Haplotype frequencies of Lr-0.7 and Lr-0.16 in stillborn piglets were significantly higher than those in live piglets (p<0.01). On the other hand, the frequencies of Lr-0.11 and Lr-0.13 in stillborn piglets were lower than those in live piglets (p<0.01). Taken together, Lr-0.7, Lr-0.16, and Lr-0.23 were associated with more stillbirths, whereas Lr-0.11 and Lr-0.13 were associated with fewer stillbirths.

**Figure 6:**
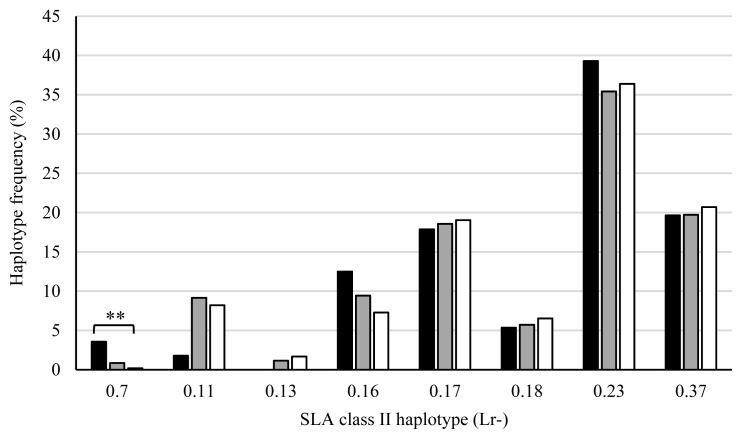
Comparison of swine leukocyte antigen (SLA) class II haplotype frequencies of sires among the three groups of litters. X-axis shows haplotypes of homozygous or heterozygous sires. ■, AS group (the number of haplotypes is 56); ■, PS group (the number of haplotypes is 350), □, AA group (the number of haplotypes is 536). ** p<0.01.

**Figure 7:**
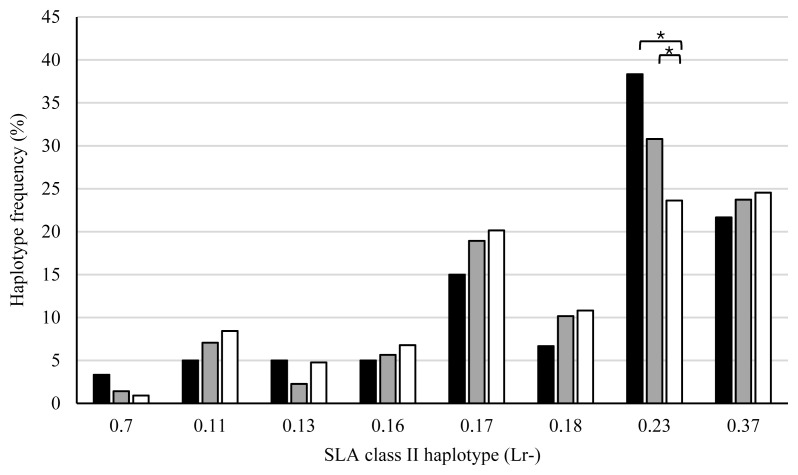
Comparison of swine leukocyte antigen (SLA) class II haplotype frequencies of dams among the three groups. X-axis shows the haplotypes of homozygous or heterozygous dams. ■, AS group (the number of haplotypes is 60), ■, PS group (the number of haplotypes is 354), □, AA group (the number of haplotypes is 546). * p<0.05.

SLA class II haplotype frequencies of sires and dams were compared among the three groups (Figures 6, 7). A significant difference (p<0.01) was observed between the frequencies of sires with Lr-0.7 in the AS and AA groups, suggesting an association between the Lr-0.7 and stillbirth. In dams, the haplotype frequency of Lr-0.23 in the AS group was significantly higher than those in the PS (p<0.05) and AA (p<0.05) groups. In addition, the frequency of dams with Lr-0.7 in the AS group were higher than those in the PS and AA group, although the differences were not statistically significant.

## DISCUSSION

The incidences of stillbirths in domesticated pigs of commercial breeds vary between 3% and 8% [1,17,18]. In our population of MMPs, the incidence of stillbirths was 17.7%, which is more than double the rate of that in pigs of commercial breeds. The relatively high incidence of stillborn piglets in MMPs seems to be associated with their extra small body sizes [13]. The average litter size was 5.48±0.04 (mean±standard error) in a population of MMPs of our previous study [16], which is almost the same value as that in the AA group (5.36± 0.12) of the present study. On the other hand, the AS group had the smallest average litter size (3.87±0.39) compared to the PS (6.38±0.14) or AA group. In contrast, large litter sizes and small body weights at birth were suggested to be risk factors for stillbirth in domesticated pigs [3,19] and that might lead to the extension of farrowing duration and induce the stillbirth [2,18,20,21]. Although the occurrence of the stillbirth might be attributed to large litter sizes, the distribution patterns of litter sizes were normal in the PS and AA groups, but not in the AS group of MMPs. Also in domestic pigs, larger stillbirth numbers were commonly associated with higher parities [2,19] and/or in the first parity dams possibly due to a narrower birth canal at the first farrowing [2,18,19]. In MMPs, the percentage of parity in each group tended to decrease gradually as the parity increased in all three groups. Therefore, we could not conclude from our findings that there was a direct relationship between the occurrence of stillbirth and parity in the AS group.

Recently, genome wide association studies (GWAS) were applied for identification of affecting regions of average birth interval and stillbirth in swine [4]. Furthermore, to identify possible candidate genes affecting average birth interval and stillbirth, GWAS were also undertaken in Landrace-Duroc-Yorkshire females by Schneider et al [4]. ADAM metallopeptidase with thrombospondin type 1 motif, 9 (*ADAMTS9*), collagen 19 (*COL19A1*), nucleotide binding protein-like (*NUBPL*), LOC100518697 (a nostrin-like gene), and dipeptidyl-peptidase 10 (*DPP10*) were identified as the candidate genes that might help in improving marker assisted selection. However, it has not been revealed how these candidate genes might be involved in the average birth interval and occurrences of stillbirth. In a more recent study using GWAS, Sanglard et al [22] showed that a single nucleotide polymorphism (SNP, H3GA0020505) on the SLA class II region was associated strongly with reproductive performance including with the number of liveborn and stillborn piglets in PRRSV vaccinated commercial sows. However, no candidate genes involving reproductive performance were identified near the SNP.

In the present study, we analyzed some associations be tween the SLA class II haplotypes and stillbirth in MMPs. Comparison of SLA class II haplotype frequencies in piglets, sires, or dams among three groups, AS, PS, and AA groups, exhibited that Lr-0.7, Lr-0.16, and Lr-0.23 were associated with more stillbirths, whereas Lr-0.11 and Lr-0.13 were associated with fewer stillbirths. In a MMP population, body weights at birth and 50 days after birth were relatively low in pigs with Lr-0.7 and Lr-0.23, and relatively high in pigs with Lr-0.13 compared to those with the other five haplotypes [15]. We also found that the two haplotypes, Lr-0.7 or Lr-0.23, were associated with high stillbirth rates. Therefore, differences in body sizes associated with SLA class II haplotypes might be involved with occurrences of stillbirth in MMPs, however, Lr-0.7 was the lowest frequency haplotype in the MMP population [16]. The same SLA class II high resolution haplotype (Hp), Hp-0.7, also was identified previously in Yucatan miniature pigs, but associations between the haplotype and reproductive traits such as number of stillbirths per delivery were not reported for this breed [23]. On the other hand, Lr-0.7 was assigned with high frequencies in both the piglets and the dams in the AS group. Also, we observed in our previous study that the number of stillbirths per delivery tended to be larger in dams and sires with Lr-0.7, although the statistically significance could not be observed between Lr-0.7 and the other seven haplotypes due to the low frequency of MMPs with Lr-0.7 [16]. Thus, the SLA class II haplotype Lr-0.7 appears to be the most strongly associated with high stillbirth numbers and poor reproductive fitness. Furthermore, pigs with Lr-0.7 might disappear from the MMPs population in the near future if specific breeding programs are not performed to conserve them. Moreover, the SLA class II genes or haplotypes might not be directly responsible for occurrence of stillborn piglets. Although the polymorphic features of SLA genes or haplotypes might correlate only indirectly with stillbirth of MMPs, it nevertheless is evident from the present study that SLA class II haplotypes could be useful genetic markers for a more effective breeding management of MMPs to produce lower rates of stillbirths.

In this study, we focused our attention on an examination of the associations between SLA class II haplotypes and stillbirth rates. In future investigations, it would be relevant to extend our present study on SLA haplotype associations by including both the MHC class I and II genomic regions and molecules in relation to stillbirth rates in the MMPs population and other pig breeds with various SLA haplotypes.

## Figures and Tables

**Table 1:** Distribution of parities in all stillborn, partial stillborn, and all alive groups

Group (Total number of deliveries in the group)		Parity																

		1	2	3	4	5	6	7	8	9	10	11	12	13	14	15	16	17
AS	No. of deliveries	8	6	0	1	3	0	4	1	2	0	0	0	0	1	1	0	0
(27)	%	29.6	22.2	0	3.7	11.1	0.0	14.8	3.7	7.4	0	0	0	0	3.7	3.7	0	0
PS	No. of deliveries	20	18	21	21	12	11	14	9	10	11	7	3	5	4	5	2	0
(173)	%	11.6	10.4	12.1	12.1	6.9	6.4	8.1	5.2	5.8	6.4	4.0	1.7	2.9	2.3	2.9	1.2	0.0
AA	No. of deliveries	35	30	29	27	19	23	22	23	19	10	9	7	5	2	2	1	1
(264)	%	13.3	11.4	11.0	10.2	7.2	8.7	8.3	8.7	7.2	3.8	3.4	2.7	1.9	0.8	0.8	0.4	0.4

AS, all stillborn; PS, partial stillborn; AA, all alive.
